# Effects of age, period, and cohort on the prevalence of frailty in Chinese older adults from 2002 to 2014

**DOI:** 10.3389/fpubh.2022.935163

**Published:** 2022-08-12

**Authors:** Siying Li, Wenye Fan, Boya Zhu, Chao Ma, Xiaodong Tan, Yaohua Gu

**Affiliations:** ^1^School of Public Health, Wuhan University, Wuhan, China; ^2^School of Nursing, Wuhan University, Wuhan, China

**Keywords:** frailty, trajectory, age-period-cohort, centenarian, social experience

## Abstract

**Background:**

Currently, longitudinal studies on frailty are in an early stage, particularly in low- and middle-income countries. Only one study was conducted in Hong Kong to examine age-period-cohort effects on the prevalence of frailty among Chinese older adults.

**Objectives:**

This study aims to shed light on the prevalence trajectory of frailty among older adults in mainland China through the APC model and to analyze the effects of age, period, and cohort on the prevalence trajectory.

**Methods:**

The sample for this study was older adults aged 65–109 years old from the 2002 to 2014 Chinese Longitudinal Healthy Longevity Survey (CLHLS). Frailty status was measured by Rockwood FI. An age-period-cohort model was used to describe the effects of age, period, and cohort on the prevalence trajectory of frailty.

**Results:**

The prevalence of frailty among Chinese older adults changed significantly with age, period, and cohort. Furthermore, the effect of age was much stronger than the effect of period and cohort. The prevalence of frailty in the 101–103 and 104–106 age groups was 8.998 (95% CI 13.667–5.924) and 8.699 (95% CI 13.037–5.805) times higher than the in the 65–67 age group, respectively. The sensitivity analysis based on Fried's frailty phenotype showed similar results, confirming the robustness of our findings.

**Conclusion:**

All of the age effect reflecting the individual aging process, period effect reflecting change in the social environment, and birth cohort effect reflecting different generations could influence the prevalence of frailty at the population level. In contrast, the age effect was the main effect.

## Introduction

Frailty, a geriatric syndrome shaped by interacting physical, psychological, lifestyle-related, and sociodemographic factors, is characterized by decreased functions of multiple physiological systems and heightened vulnerability to stressors (1). Frailty is related to a broad range of adverse outcomes such as declining healthy life expectancy (2) and increasing healthcare expenditure (3). With rapid expansion of the aging population, frailty is becoming one of the significant global public health challenges (4).

The reported longitudinal studies have provided some valuable yet controversial information on the frailty trajectory. Several longitudinal studies suggested that frailty is dynamic, which means that the severity of frailty can deteriorate or be ameliorated with aging (4, 5). Other longitudinal studies reported that frailty levels increase with aging (6). Furthermore, some studies suggest that frailty is stable across birth cohorts (7). The latter cohort showed higher frailty levels than their predecessors at the same age (2, 8, 9). Therefore, it is crucial to conduct more longitudinal studies to gain an insight into the secular trajectory of frailty.

The age-period-cohort (APC) model is a classic demography and epidemiological tool mainly used to describe and analyze morbidity and mortality trends (10, 11). The age, period, and cohort effects are all time-related but carry different substantial implications. The age effect encompasses physiological changes and accumulation of social experience as individuals age. The cohort effect is defined as a change in morbidity across generations that have different historical experiences. The period effect represents a variation over time, simultaneously affecting all age groups and birth cohorts (12, 13). Consequently, the APC model can not only describes secular trends in diseases by assessing the independent effects of age, period, and birth cohort on mortality or morbidity but also derives clues from them about broader social, economic, and cultural context influences (12).

To our knowledge, longitudinal studies on frailty are in an early stage and have predominantly been conducted in high-income countries. So far, only a single study from Hong Kong described the age-period-cohort effects on frailty trajectory among Chinese older adults (8). Because of the complex historical background of China, more studies are needed. This study aims to understand how age, period, and cohort affect work on the trajectory of frailty prevalence among older adults from mainland China.

## Methods

### Samples

**Figure 1:**
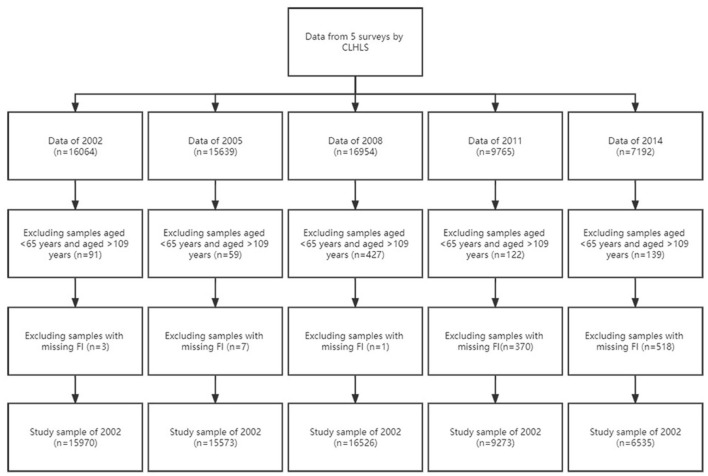
Flowchart of the study population.

Data for this study were obtained from the Chinese Longitudinal Healthy Longevity Survey (CLHLS), which is a repeated cross-sectional survey. Between 1998 and 2018, CLHLS was conducted eight times by interviewing older adults in half of randomly selected counties and cities in 23 provinces/municipalities/autonomous regions in China. Since 2005, those who died and lost older adults are being replaced by a sample of the same gender and age, and since 2011, no new replacement respondents are being added, just older adults who had been interviewed in the last survey and were still alive, and relatives of those who had died after being interviewed in the last survey. CLHLS has the most extensive samples of older adults in the world's longitudinal surveys (14, 15). The survey has been tested by many research studies and is generally acknowledged by scholars nationally and internationally (16). A comprehensive questionnaire was used to collect information on fundamental data, activities of daily living (ADLs), psychological and physical characteristics, etc. As the survey contents of the 1998 and 2000 surveys were insufficient to calculate the frailty index (FI), the data of the 2018 survey did not qualify for the APC model. The study used data from the 2002 to 2014 waves. After excluding older adults aged < 65 years old and those aged > 109 years old, the sample sizes for 2002 to 2014 were 15,970, 15,573, 16,526, 9,273 and 6,535, respectively (Figure 1).

### Measures

The Rockwood FI, defined as the proportion of deficits to all potential deficits for an individual, was a stable and reliable measure of frailty for CLHLS (15, 17, 18). We calculated the FI with 39 health metrics from various dimensions, including cognitive function (measured by Mini-Mental State Examination), chronic illness (hypertension, diabetes, tuberculosis, cardiopathy, stroke/cerebrovascular disease, bronchitis/asthma, cancer, arthritis, bedsores, gastro/duodenal ulcer, and Parkinson's disease), ADLs (eating, bathing, dressing, use of a toilet, indoor activities, continence, visiting neighbors' homes, shopping, cooking, washing laundry, walking 1 km in a row, lifting a bag weighing 5 kg, squatting and standing up three times in a row, traveling by transport), bodily function (hand behind neck, hand behind the lower back, arms up, able to stand up from sitting, and able to pick up books from the floor), psychological status (feeling fearful/anxious, feeling lonely, and feeling useless because of age), hearing ability, visual function, self-reported health, and others (cardiac rhythm, interviewer-rated health, and frequency of serious illness in the last 2 years) (19). FI ≥ 0.25 means frailty (20).

### Age–period–cohort analysis

For the APC analysis, the data in this study were collated as 15 successive three-year age groups ranging from 65–67 years to 107–109 years, five periods (2002, 2005, 2008, 2011, and 2014), and 19 three-year cohorts ranging from 1893–895 to 1947–1949. Since the equation cohort = period–age, there was a significant covariance between the age, period, and cohort effects, making it difficult to analyze their independent effects (21). The Intrinsic Estimator (IE), which has unbiasedness, validity, progressivity, and superior estimation ability, was used to solve this problem (22, 23).

### Sensitivity analysis

For sensitivity analysis, we also defined the frailty status based on frailty phenotype (modified Fried criteria), another widely used frailty measurement tool. Fried's criteria included five domains, which were exhaustion (feeling useless because of age), shrink (BMI <18.5 kg/m^2^), weakness (unable to lift a bag weighing 5 kg), low mobility (unable to walk 1 km in a row), and inactivity (participate in the following activities one time per week or less: housework, outside activity, growing flower or pet, keeping poultry or domestic animals or livestock breeding, playing cards or mah-jongg, and social activity) (24). Height was lacking in the 2002 data, so we used total arm length (length from wrist to shoulder) to estimate it (25). Older adults were considered frail if they featured ≥3 domains (26).

### Statistical analysis

We performed APC analysis using STATA 16.0 and plotted a heatmap using the ggplot2 and pheatmap packages in R v.4.1.2. *P* < .05 indicated statistical significance.

## Results

### Descriptive analysis

**Figure 2:**
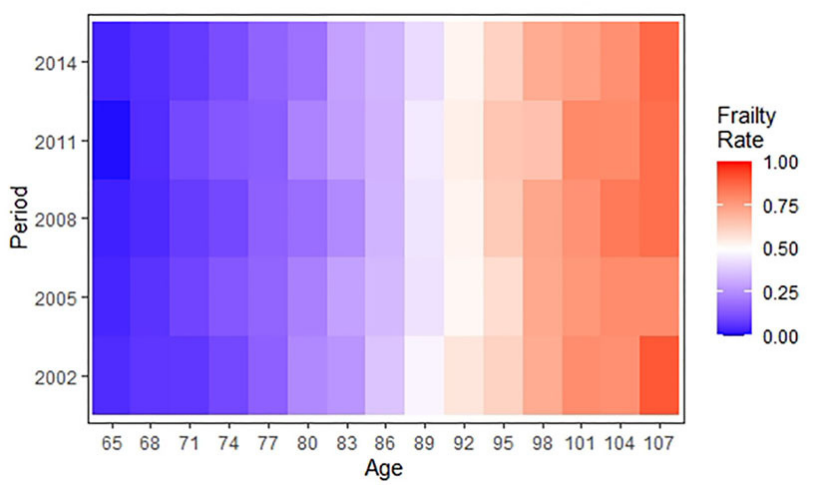
A heatmap showing the age-period distribution of frailty rates among Chinese older adults during 2002–2014.

Table 1 shows the essential characteristics of participants in 5 waves between 2002 and 2014. The gender and age distributions were broadly similar in each cross-section. The number of women was slightly larger than the number of men, with essentially the largest sample size in the 80–89 age group and the smallest sample size in the 65-69 age group. Taking the data of 2002 as an example, 42.7% were men, 10.1% were 65–69 years old, 20.3% were 70–79 years old, 26.5% were 80–89 years old, 23.5% were 90–99 years old, and 19.7% were 100–109 years old. In addition, comparing the prevalence of frailty by period shows the highest prevalence of frailty in 2014 (65.3%) and the lowest prevalence of frailty in 2008 (59.5%). Figure 2 further shows the distribution of prevalence of frailty among older adults by period and age group. As shown in the graph, the prevalence of frailty continuously increased with aging but showed no clear changes over periods in the same age group.

**Table 1:** Basic characteristics of the sample.

	**2002** **(*n =* 15,970)**	**2005** **(*n =* 15,573)**	**2008** **(*n =* 16,526)**	**2011** **(*n =* 9,273)**	**2014** **(*n =* 6,535)**
**Sex**					
Male	6,817 (42.7)	6,675 (42.9)	6,986 (42.3)	4,186 (45.1)	2,989 (45.7)
Female	9,153 (57.3)	8,898 (57.1)	9,540 (57.7)	5,087 (54.9)	3,546 (54.3)
**Age (year)**					
65–69	1,608 (10.1)	1,671 (10.7)	1,402 (8.5)	635 (6.8)	208 (3.2)
70–79	3,237 (20.3)	3,281 (21.1)	2,884 (17.5)	2,439 (26.3)	2,003 (30.7)
80–89	4,236 (26.5)	3,907 (25.1)	4,278 (25.9)	2,543 (27.4)	2,045 (31.3)
90–99	3,747 (23.5)	3,950 (25.4)	4,620 (28.0)	2,321 (25.0)	1,516 (23.2)
100–109	3,142 (19.7)	2,764 (17.7)	3,342 (20.2)	1,335 (14.4)	763 (11.7)
**Frailty**					
Yes	9,626 (60.3)	9,589 (61.6)	9,798 (59.3)	5,809 (62.6)	4,268 (65.3)
No	6,344 (39.7)	5,984 (38.4)	6,728 (40.7)	3,464 (37.4)	2,267 (34.7)

**Figure 3:**
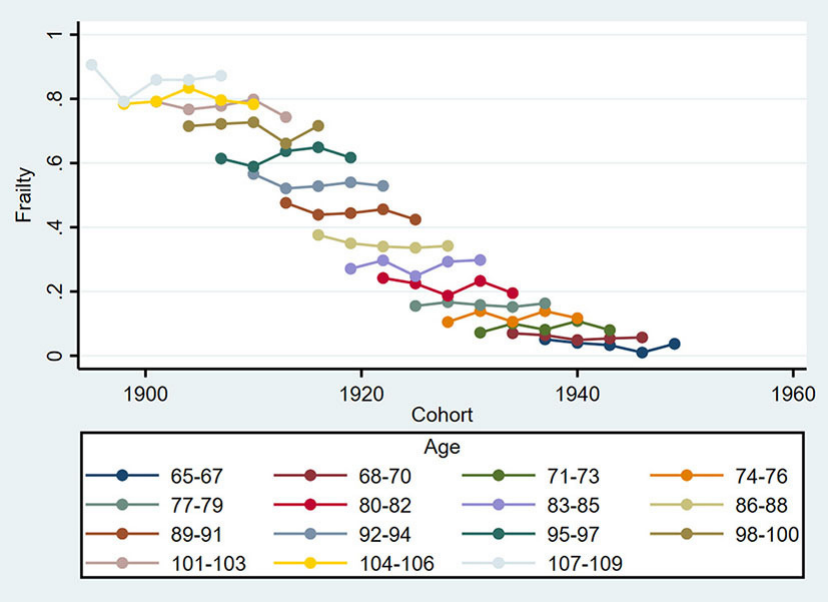
Frailty rates by birth cohort and age group among Chinese older adults during 2002–2014.

The age-specific prevalence at intervals of 3 years of age for frailty is plotted against the birth cohort in Figure 3. The prevalence of frailty increased distinctly with aging in same birth cohorts while varying irregularly with birth cohort in same age groups. Concretely, in the 65–70, 80–82, 86–94, and 101–109 age groups, the prevalence of frailty was lower in the more recent cohorts than in the earlier cohorts. Although Figures 2, 3 confound the age, period, and cohort effects, it can be tentatively judged that the prevalence of frailty in older adults in the same age group differed across birth cohorts and periods.

### Age–period–cohort analysis with the intrinsic estimator

**Figure 4:**
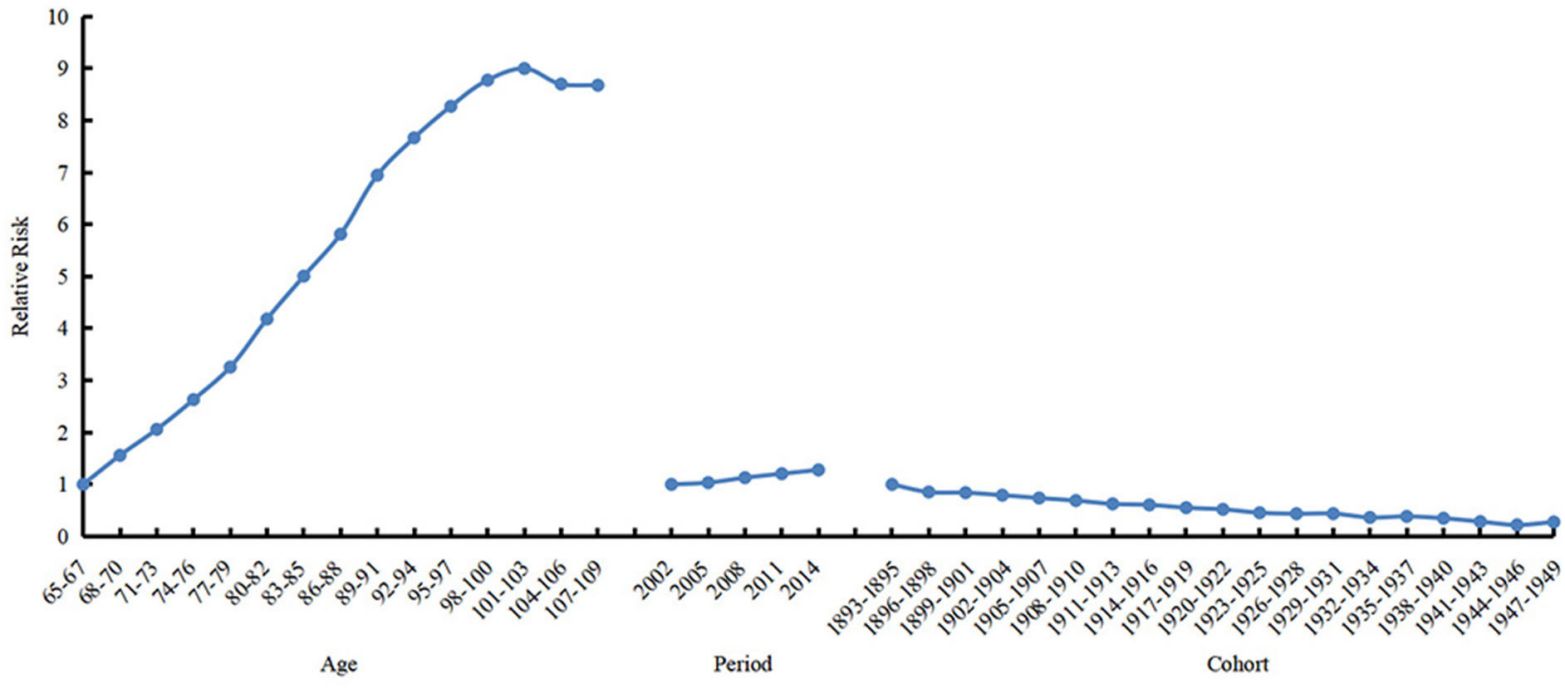
Relative risks of age (left), period (mid) and cohort (right) effects on prevalence of frailty.

The 69–71 years old, 2002, period, and 1893s−1895s cohort were selected as references to calculate estimated relative risks of the frailty incidence for other age, period, and birth cohort groups, respectively. The results are shown in Table 2 and Figure 4. The age effect increased approximately linearly with aging from the 65 age group to the 103 age group, and decreased slightly from the 104 age group to the 109 age group. The prevalence of frailty in the 101–103 and 104–106 age groups was 8.998 (95% CI 13.667–5.924) and 8.699 (95% CI 13.037–5.805) times higher than in the 65–67 age group, respectively. The period effect increased slightly over time. The prevalence of frailty in 2014 was 1.279 (95% CI 1.281–1.277) times higher than in 2002. The cohort effect generally decreased from 1895s to 1951s. The prevalence of frailty in the 1947s−1949s birth cohort was .277 (95% CI 0.109–0.705) times higher than in the 1893s−1895s cohort.

**Table 2:** Results of the APC model of prevalence of frailty among Chinese older adults during 2002–2014.

**Age**	**RR (95% CI)**	**Period**	**RR (95% CI)**	**Cohort**	**RR (95% CI)**
65–67	1.000^**^	2002	1.000^**^	1893–1895	1.000^**^
68–70	1.559 (1.915–1.269)^**^	2005	1.034 (1.051–1.018)^**^	1896–1898	0.854 (0.870–0.838)^**^
71–73	2.059 (2.704–1.567)^**^	2008	1.129 (1.156–1.103)	1899–1901	0.841 (0.870–0.813)^**^
74–76	2.630 (3.586–1.928)^**^	2011	1.206 (1.225–1.187)^**^	1902–1904	0.793 (0.829–0.758)^**^
77–79	3.256 (4.548–2.332)^**^	2014	1.279 (1.281–1.277)^**^	1905–1907	0.738 (0.777–0.702)^**^
80–82	4.181 (5.970–2.928)			1908–1910	0.689 (0.724–0.656)^**^
83–85	5.006 (7.289–3.437)			1911–1913	0.626 (0.652–0.600)^**^
86–88	5.812 (8.602–3.927)^**^			1914–1916	0.606 (0.625–0.588)^**^
89–91	6.946 (10.447–4.618)^**^			1917–1919	0.552 (0.561–0.544)
92–94	7.665 (11.673–5.033)^**^			1920–1922	0.521 (0.521–0.520)
95–97	8.270 (12.683–5.393)^**^			1923–1925	0.455 (0.447–0.464)
98–100	8.775 (13.443–5.727)^**^			1926–1928	0.437 (0.422–0.452)^*^
101–103	8.998 (13.667–5.924)^**^			1929–1931	0.438 (0.417–0.461)
104–106	8.699 (13.037–5.805)^**^			1932–1934	0.365 (0.337–0.395)^*^
107–109	8.676 (12.796–5.883)^**^			1935–1937	0.385 (0.350–0.423)^*^
				1938–1940	0.349 (0.304–0.401)^*^
				1941–1943	0.285 (0.223–0.364)^*^
				1944–1946	0.222 (0.133–0.369)^*^
				1947–1949	0.277 (0.109–0.705)
Deviance			0.014
AIC			−4.806
BIC			−168.370

* p < 0.05,

** p < 0.001. RR, relative risk; CI, confidence interval; AIC, Akaike Information Criterion; BIC, Bayesian Information Criterion.

### Sensitivity analysis

**Figure 5:**
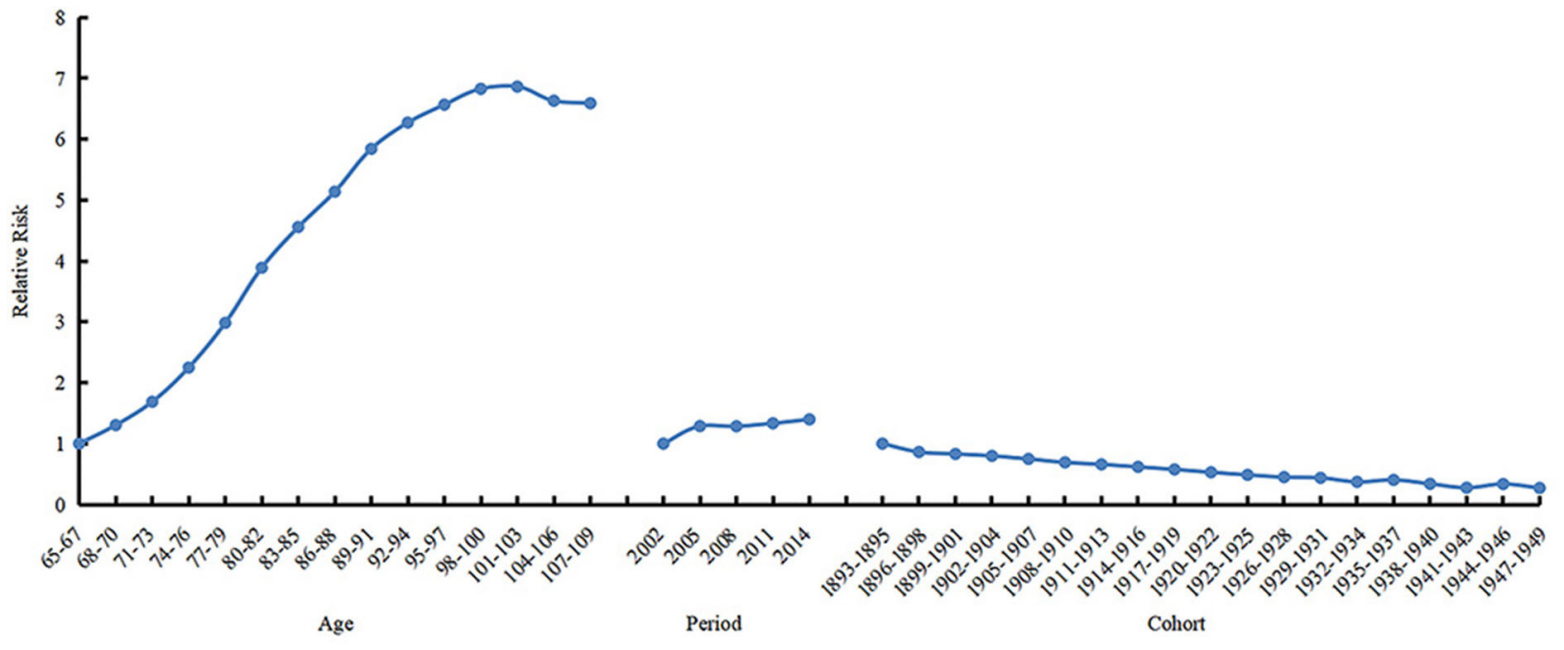
Sensitivity analysis with the frailty measured by frailty phenotype: Relative risks of age (left), period (mid) and cohort (right) effects on prevalence of frailty.

The results were similar when APC analysis was performed with the frailty measured by frailty phenotype (Table 3; Figure 5). The prevalence of frailty in the 101–103 and 104–106 age groups was 6.863 (95% CI 9.264–5.085) and 6.632 (95% CI 8.856–4.966) times higher than in the 65–67 age group, respectively. The prevalence of frailty in 2014 was 1.398 (95% CI 1.408-1.389) times higher than in 2002. The prevalence of frailty in the 1947s−1949s birth cohort was .272 (95% CI 0.135–0.546) times higher than in the 1893s−1895s birth cohort.

**Table 3:** Sensitivity analysis with frailty measured by frailty phenotype: Results of the APC model of prevalence of frailty among Chinese older adults during 2002–2014.

**Age**	**RR (95% CI)**	**Period**	**RR (95% CI)**	**Cohort**	**RR (95% CI)**
65–67	1.000^**^	2002	1.000^**^	1893–1895	1.000^**^
68–70	1.304 (1.425–1.193)^**^	2005	1.288 (1.308–1.268)	1896–1898	0.863 (0.925–0.805)^**^
71–73	1.687 (1.919–1.483)^**^	2008	1.285 (1.307–1.264)	1899–1901	0.831 (0.924–0.747)^**^
74–76	2.249 (2.656–1.905)^**^	2011	1.334 (1.352–1.317)	1902–1904	0.798 (0.909–0.701)^**^
77–79	2.984 (3.617–2.462)^*^	2014	1.398 (1.408–1.389)^**^	1905–1907	0.748 (0.863–0.648)^**^
80–82	3.889 (4.803–3.148)			1908–1910	0.692 (0.792–0.604)^**^
83–85	4.558 (5.731–3.625)			1911–1913	0.659 (0.745–0.583)^*^
86–88	5.138 (6.568–4.020)^*^			1914–1916	0.618 (0.687–0.556)^*^
89–91	5.842 (7.609–4.485)^**^			1917–1919	0.576 (0.628–0.528)
92–94	6.273 (8.302–4.739)^**^			1920–1922	0.529 (0.566–0.495)
95–97	6.567 (8.809–4.896)^**^			1923–1925	0.487 (0.511–0.463)
98–100	6.827 (9.228–5.051)^**^			1926–1928	0.450 (0.464–0.436)
101–103	6.863 (9.264–5.085)^**^			1929–1931	0.438 (0.445–0.431)
104–106	6.632 (8.856–4.966)^**^			1932–1934	0.372 (0.369–0.375)^*^
107–109	6.591 (8.611–5.044)^**^			1935–1937	0.404 (0.399–0.409)
				1938–1940	0.340 (0.316–0.366)^*^
				1941–1943	0.276 (0.230–0.330)^*^
				1944–1946	0.337 (0.259–0.438)
				1947–1949	0.272 (0.135–0.546)
Deviance			0.146
AIC			6.287
BIC			−162.670

* p < 0.05,

** p < 0.001. RR, relative risk; CI, confidence interval; AIC, Akaike Information Criterion; BIC, Bayesian Information Criterion.

## Discussion

Our study explored the frailty trajectory of Chinese older adults aged 65–109 years from 2002 to 2014 using the APC model. The age effect was proven to be the most obvious effect on the prevalence of frailty. In addition, the earlier cohorts and closer periods had higher frailty prevalence, although the magnitude of the effects was considerably weaker than that of aging.

Consistent with previous studies, the prevalence of frailty increased substantially with aging in older adults aged 65 to 103 years in this study. Furthermore, we found a slight downward trend in the prevalence of frailty for older adults over 104 years old. The available evidence suggested that centenarians usually have an exceptionally healthy aging phenotype (27), which may be attributed to their exceptional genetic profiles, healthy lifestyle, and open-mindedness (28, 29). For example, Belenguer-Varea et al. found that compared to the general elderly population, centenarians exhibited less oxidative damage, particularly lower plasma lipid peroxidation biomarkers (30). Franca Rosa et al. suggested that centenarians had an unusual colony of cytotoxic CD4+ T cells under the effect of a human leukocyte antigen (31). Arai et al. found that maintenance of insulin sensitivity, low prevalence of diabetes, and dysregulation of adipokines were characteristics of centenarians (32). All of these features help to enhance immunity, reduce inflammation, and prevent frailty. In addition, Julia et al. found that apolipoprotein levels were decreased significantly with aging but generally shifted to higher levels in centenarians, with ApoB, ApoC3, and ApoE being positively correlated with cognition (33).

The increased prevalence of frailty in older adults from 2002 to 2014 might have resulted from population aging. According to the Chinese National Census, the percentage of the population aged more than 65 years increased from 6.96% in 2000 to 8.87% in 2010. With the development of society and medical science, humankind's surveillance rate and life expectancy have increased remarkably. Meanwhile, it directly contributed to the growth of the aging population and prolongation of unhealthy living states.

1893s−1949s was a long and harsh wartime in Chinese history, spanning the Siege of the International Legations, the downfall of the Qing Dynasty, World War II (WWII), and the founding of New China (34, 35). Our study suggested that younger cohorts born during this period had lower prevalence of frailty than the older cohorts. Several studies from Finland and Vietnam have also shown a positive correlation between exposure to potentially traumatic wartime experiences in early life and frailty in later life in the sense that war promotes frailty (36–38). However, the unique social structures of each region may lead to completely opposite conclusions. For example, in the early to mid-20th century, Hong Kong enjoyed relative social stability and economic prosperity compared to the wartime state of the mainland, which resulted in the mid-1900s birth cohorts having more extended life expectancy and higher levels of frailty than cohorts born in the early 1900s (8). In addition, A German-based study also found that cohorts born during WWII (1945s−1948s) had lower FI levels than cohorts born after WWII (1948s−1950s). It may be attributable to the fact that Germany, as the initiator of WWII, did not deteriorate nutritionally during the war but experienced early reconstruction and a severe food crisis after its defeat (39). Thus, we argue that in addition to the direct physical damage caused by the war and social unrest, its concomitant poverty, nutritional and medical resource scarcity, house destruction, poor living conditions, and mental trauma all have adverse long-term effects on health. Furthermore, these traumas, when occurring in early and mid-life (especially critical developmental age), can lead to sustained accumulation of stress and contribute to frailty in later life by affecting genetic stability, cellular function, and inflammatory susceptibility (38).

This study provides an important and reliable addition to extant research studies on frailty. First, this study used data from CLHLS, which is highly representative of Chinese older adults. Second, this study is the first longitudinal study examining the age, period, and cohort effects on frailty trajectory among older adults in mainland China. Third, the older adults in this study have lived through war at varying degrees, facilitating an insight into the impact of social unrest on frailty in developing countries. Fourth, the proportion of centenarians in this study is relatively high, which can provide a valuable insight into the age effect on frailty. Fifth, we used the two most widely used frailty indicators (i.e., frailty phenotype and FI) to enhance the reliability of the results. The study has several limitations. First, it is possible that some frail individuals did not survive in the age and period included in the study, inevitably leading to survival bias that may underestimate the prevalence of frailty. Second, the FI contains numerous chronic disease and ADL variables that are difficult to rehabilitate or reverse, which may falsely enhance the increasing effect of age on frailty. Finally, this study did not include older adults in remote areas of China, and the recent history of China is quite specific, both of which may limit the generalization of our findings.

In conclusion, all of the age effect reflecting the individual aging process, period effect reflecting change in social environment, and birth cohort effect reflecting different generations could significantly influence the prevalence of frailty at the population level. Among the three effects, age was the main effect, and the prevalence of frailty increased significantly along with individual aging. This is in accordance with current knowledge and evidence of aging and frailty. Moreover, it is worth noting that the prevalence of frailty was also influenced by the period effect and birth cohort effect. According to our results, lower prevalence of frailty was observed in more recent cohorts, which provided evidence for the impact of social and environmental factors on the development of frailty at the population level. However, further studies are needed to explore the underlying mechanisms.

## Data availability statement

The datasets presented in this study can be found in online repositories. The names of the repository/repositories and accession number(s) can be found below: Chinese Longitudinal Healthy Longevity Survey (CLHLS), https://www.icpsr.umich.edu/web/NACDA/studies/36692/datadocumentation.

## Ethics statement

This study was a secondary analysis using CLHLS data and was approved by the Research Ethics Committee of Peking University (IRB00001052-13074). All the participants or their proxy respondents provided written informed consent.

## Author contributions

YG designed the study and revised the manuscript. SL analyzed the data and drafted the manuscript. XT critically revised the manuscript. WF, BZ, and CM contributed to the interpretation of data. All the authors read and approved the manuscript.

## Conflict of interest

The authors declare that the research was conducted in the absence of any commercial or financial relationships that could be construed as a potential conflict of interest.

## Publisher's note

All claims expressed in this article are solely those of the authors and do not necessarily represent those of their affiliated organizations, or those of the publisher, the editors and the reviewers. Any product that may be evaluated in this article, or claim that may be made by its manufacturer, is not guaranteed or endorsed by the publisher.
